# Supplementary material for: Is it safe to nest near conspicuous neighbours? Spatial patterns in predation risk associated with the density of American Golden-Plover nests
Source: PeerJ. 2016 Aug 10;4:e2193. doi: 10.7717/peerj.2193 (PMC4991854; doi:10.7717/peerj.2193)
Supplement: Table S1 — We compared models including up to four of the following predictors: distance in metres to the associated plover nest (Distance), distance in metres to the closest active plover nest (Active Distance), density of active American Golden-Plover nests within a radius of 250 m around the artificial nest (Active Density), habitat type, i.e. wetland or mesic tundra (Moisture) and vertical concealment (Concealment). We also tested for quadratic effects of Distance and Active Distance. We report Akaike’s information criterion corrected for small sample size (AICc), difference in AICc relative to the model with the lowest AIC (ΔAICc), as well as the AICc weight (ωAICc). Models are ranked by their AICc values and the best-fitting models (ΔAICc >2) are shown in bold. [file peerj-04-2193-s002.docx]

**Is it safe to nest near conspicuous neighbours? Spatial patterns in predation risk associated with the density of American Golden-Plover nests**

Marie-Andrée Giroux, Myriam Trottier-Paquet, Joël Bêty, Vincent Lamarre and Nicolas Lecomte

# Supplemental Table

Table S1. Selection of models explaining variations in the risk of predation of artificial nests over 12 exposure days on Igloolik Island (Nu, Canada) during the summer of 2014 (*n*=24). We compared models including up to four of the following predictors: distance in metres to the associated plover nest (Distance), distance in metres to the closest active plover nest (Active Distance), density of active American Golden-Plover nests within a radius of 250 m around the artificial nest (Active Density), habitat type, i.e. wetland or mesic tundra (Moisture) and vertical concealment (Concealment). We also tested for quadratic effects of Distance and Active Distance. We report Akaike’s information criterion corrected for small sample size (AICc), difference in AICc relative to the model with the lowest AIC (ΔAICc), as well as the AICc weight (ωAICc). Models are ranked by their AICc values and the best-fitting models (ΔAICc < 2) are shown in bold.

| Model | AICc | ΔAICc | ωAICc |
| --- | --- | --- | --- |
| **Active Density** | **1237.0** | **0.00** | **0.36** |
| **Active Density + Moisture** | **1239.0** | **1.99** | **0.13** |
| Active Density + Concealment | 1239.0 | 2.04 | 0.13 |
| Active Distance | 1239.3 | 2.36 | 0.11 |
| Active Distance + Active Distance^2^ | 1240.3 | 3.38 | 0.07 |
| Active Density + Concealment + Moisture | 1241.1 | 4.10 | 0.05 |
| Active Distance + Concealment | 1241.2 | 4.27 | 0.04 |
| Active Distance + Moisture | 1241.4 | 4.42 | 0.04 |
| Active Distance + Active Distance^2^ + Concealment | 1242.4 | 5.40 | 0.02 |
| Active Distance + Active Distance^2^ + Moisture | 1242.4 | 5.47 | 0.02 |
| Active Distance + Concealment + Moisture | 1243.3 | 6.37 | 0.01 |
| Active Distance + Active Distance^2^ + Concealment + Moisture | 1244.5 | 7.52 | 0.01 |
| Distance + Moisture | 1246.3 | 9.38 | 0.00 |
| Distance | 1247.8 | 10.84 | 0.00 |
| Null | 1248.1 | 11.17 | 0.00 |
| Distance + Distance^2^ + Moisture | 1248.2 | 11.25 | 0.00 |
| Distance + Concealment + Moisture | 1248.4 | 11.46 | 0.00 |
| Moisture | 1248.5 | 11.49 | 0.00 |
| Distance + Concealment | 1249.2 | 12.20 | 0.00 |
| Concealment | 1249.7 | 12.76 | 0.00 |
| Distance + Distance^2^ | 1249.8 | 12.85 | 0.00 |
| Distance + Distance^2^ + Concealment + Moisture | 1250.3 | 13.34 | 0.00 |
| Concealment + Moisture | 1250.5 | 13.57 | 0.00 |
| Distance + Distance^2^ + Concealment | 1251.2 | 13.57 | 0.00 |
